# The Surgical Teams’ Perception of the Effects of a Routine Intraoperative Pause

**DOI:** 10.1007/s00268-016-3632-9

**Published:** 2016-07-14

**Authors:** Sofia Erestam, Eva Angenete, Kristoffer Derwinger

**Affiliations:** 1 Department of Surgery, Institute of Clinical Sciences, SSORG - Scandinavian Surgical Outcomes Research Group, Sahlgrenska Academy at University of Gothenburg, Sahlgrenska University Hospital/Östra, Paviljong 11, Journalvägen 14a, 416 50 Gothenburg, Sweden; 2 Department of Surgery, Institute of Clinical Sciences, Sahlgrenska Academy at University of Gothenburg, Sahlgrenska University Hospital/Östra, Diagnosvägen 11, 416 50 Gothenburg, Sweden

## Abstract

**Background:**

A pause routine may reduce stress and errors during surgery. The aim of this study was to explore how the team, divided into the different professional groups, perceived the implementation of a pause routine and its possible impact on safety.

**Methods:**

A pause routine was introduced at a University hospital operating theatre in Sweden in 2013. Questionnaires were distributed about 1 year later to all members of the operating theatre team. The questions included different perspectives of possible effects of the pause routine.

**Results:**

A majority were positive to scheduled pauses. The surgeons often felt refreshed and at times changed their view on both anatomy and their surgical strategy. They were also perceived by other team members as improved regarding communication. All groups felt that patient safety was promoted. There were differences by profession in perception of team communication.

**Conclusions:**

The pause routine was well perceived by the surgical team. A majority believed that scheduled and regular pauses contribute to improved patient safety and better team communication. There were also findings of differences in communication and experience of team coherence between personnel categories that could benefit from further acknowledgement and exploration.

## Introduction

A surgical procedure is as a team effort requiring both focus and presence, which must be maintained during the entire procedure regardless of its length [1, 2]. There are several factors that may have a negative impact on the team’s focus during surgery, including communication failures, environmental factors, disturbances by other personnel and technical problems, all of which may increase stress [3–9]. Stress may impair the surgical performance both at a technical and a cognitive level [10]. Acute stress has been recognized as detrimental for teamwork and may thus affect the teamwork in the operating theatre and indirectly the patient safety [11, 12]. For longer procedures, it is plausible that both physical and mental strain could contribute to stress and gradually lead to fatigue. Therefore, longer operations may benefit from an extra focus on how to handle stress and fatigue to improve patient safety.

There has been an increased interest in later years in the surgeons’ non-technical skills, including leadership and communication [13, 14]. Stress handling is also of importance for the surgeon. Acknowledging and coping with fatigue and stress are important. Fatigue has been recognized as a risk to patient safety through effects on cognitive performance, motor skills, communication and social skills [3, 13, 15]. It has been suggested that individual team members can increase their awareness of their own responses to stress and how to counteract this response [10, 11, 16]. Stress can be reduced by improvements of the operating theatre environment such as noise reduction [17–19], improved communication [14, 20], team building and training [21].

Intraoperative pauses also reduce stress, as described by Engelmann et al., where a strict pause discipline significantly reduced both the stress levels and the number of errors without prolonging the operating time [22]. The pause must be initiated before fatigue is evident, and this requires the setting of pause standards. The concept of scheduled pauses has many origins. It has been described from mountain expeditions where the Sherpa tradition of pacing and pause helped them reach their destinations faster than westerners going on until tired before resting. The principle is also applied by many armed forces. The aim of taking shorter pauses is to minimize the accumulation of fatigue and ameliorate ergonomics.

As an initiative to improve patient safety and surgical teamwork, a pause routine was introduced at the unit for colorectal surgery in a University Hospital in 2013. As the operation is seen as a team effort, all staff members were included in the implementation and evaluation of this routine.

The aim of this study was to explore how the team, divided into different professional categories, perceived the pause routine itself and its’ implementation. The secondary aim was also to evaluate whether the different team members perceived that the pause had effects on the operation, the surgeon and team communication.

## Materials and methods

### Setting

The study was conducted at the Department of Surgery and the Department of Anaesthesiology at the Sahlgrenska University Hospital/Östra in Sweden. The Department of Surgery at Sahlgrenska University Hospital is a tertiary referral centre for colorectal disease, and subsequently advanced surgical procedures within both colorectal cancer and inflammatory bowel disease are performed. These procedures are often lengthy; the operating time is about 4–6 h or longer.

In 2013 a pause routine was introduced for colorectal surgical procedures and consisted of pauses every other hour, with 2 dl of liquid refreshment and a short mental break of 1–2 min, and after every 4 h a longer pause with a snack or quick lunch. In addition, pauses were encouraged after the resolution of a major adverse event or when in doubt of continued surgical strategy. The anaesthetist nurses, scrub nurses and the circulating nurses were all informed that they should ask the surgeons every 2 h if they were ready for a short pause. The requirement for taking a pause with a short snack i.e. leaving the operating theatre was a fully stable patient. The normal operating theatre staffing at this institution was two surgeons, one scrub nurse, one anaesthetist nurse and one circulating nurse, and all but the operating surgeon remained in the operating theatre during the pause. The anaesthesiologists are responsible for several operations at a time and are not normally present in the operating theatre during the procedure and were thus not included in this study.

Prior this routine, all pauses were at the individual’s initiative.

The surgical department also includes emergency surgery, upper gastrointestinal and abdominal wall surgery, but with shorter operating times, and these procedures were therefore not directly involved in this routine.

### Study design

Questionnaires were handed out to all personnel categories of the operating theatre team involved in the intraoperative pause routine. The first question in the questionnaire offered personnel a chance to answer: “I am rarely active in this type of surgical procedures where pauses are current and therefore choose to not respond to the survey.”

There were 16 surgeons, 19 scrub nurses, 34 anaesthetist nurses and 28 circulating nurses involved in, at least partly, the colorectal surgical team. The questions were constructed with four different response options: yes always; yes sometimes; no; and I don’t know. Most questions were similar for all personnel categories, however a few questions differed. One question regarding whether the understanding of the anatomy was affected was only included in the questionnaires to the surgeons and scrub nurses as it was considered impossible for the rest of the personnel to elaborate on this subject not being close to the surgical field. Furthermore, the questions “do you experience that proposals to take a pause are received in a positive manner?”, and a question about the communication with the surgeons were not addressed to the surgeons.

There was a last open-ended question giving participants the possibility of expressing their views on the subject and providing ideas of improvement.

The questionnaires were handed out after an informative meeting regarding the study at two staff meetings. After the meetings, the questionnaires were placed in the staffs’ pigeonholes at the hospital. The questionnaires could be answered under full anonymity. Operation times were attained from data registries for major surgical procedures, such as rectal cancer, both before (2011) and after (2014) the introduction of the pause routine.

### Statistical analysis

The IBM SPSS Statistics 22 software package was used for analysis. Descriptive statistics were used for analysis of the questionnaires. Independent sample *t* test was used to assess differences in operation time.

## Results

**Fig. 1:**
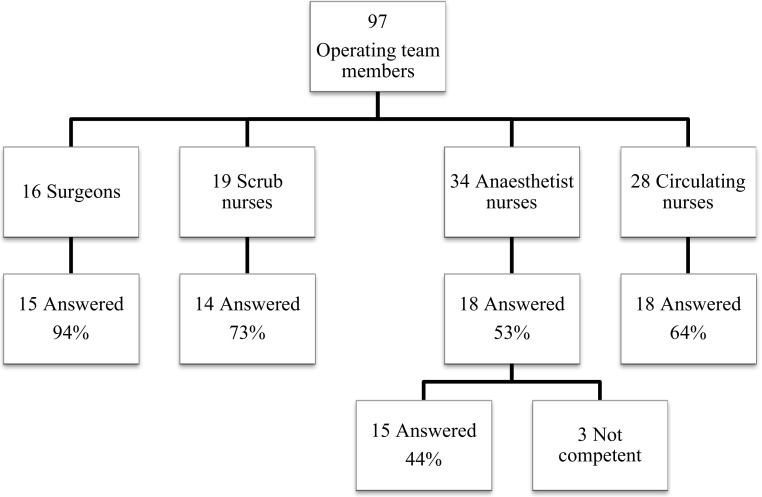
Flowchart on distributed questionnaires. Note that the surgeons were all consultants at the colorectal surgery section of the department of surgery and thus involved in major surgery. The nurses of the department of anaesthesiology and operation also work towards other surgical departments with other types of surgery

**Fig. 2:**
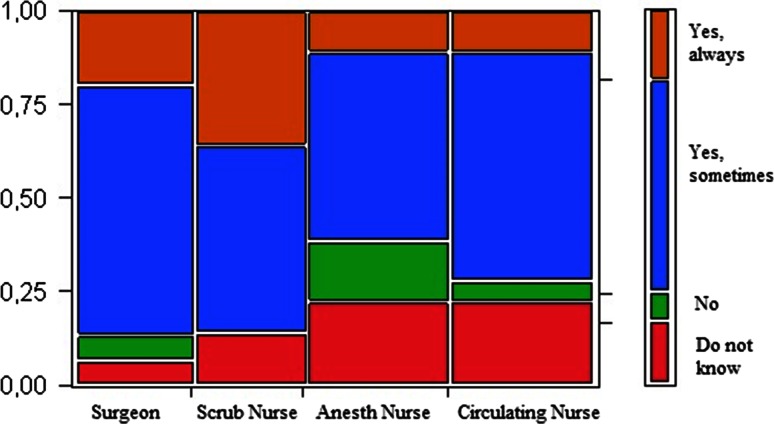
Team Work. Answers in percentages by professional category to the question “Do you perceive that the team work is better after a pause?”

The questionnaire response rate divided by professional categories is shown in Fig. 1. Many, and foremost surgeons and scrub nurses, indicated that they believed that pauses improved the team’s collaboration (Fig. 2). Correspondingly, 93 % of scrub nurses perceived it easier to communicate with surgeons after pauses, but this perception was less common among the anaesthetist nurses (61 % (11/18)) and circulating nurses (72 % (13/18)).

**Fig. 3:**
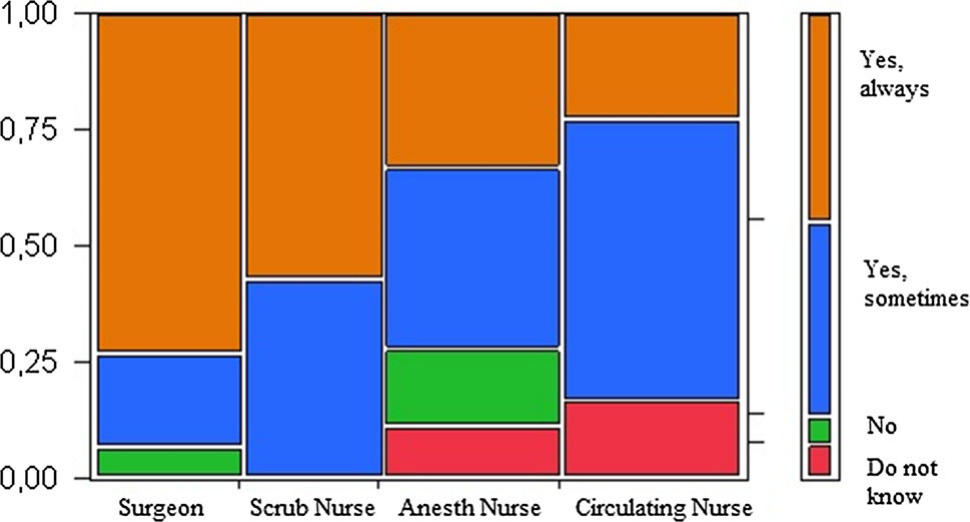
Alert after pause. Answers in percentages by professional category to the question “Do you perceive that you/the surgeon is more alert after a pause?”

The surgeons stated that they often remembered to take a pause; however, the nurses’ view was that the surgeons needed to be reminded. The scrub nurse was most frequent in reminding/suggesting pauses followed by the circulating nurse. Two scrub nurses had experienced negative feedback on suggestions of a pause, but a majority of the nurses (92 %) only received positive comments. Two surgeons had a routine of planning pauses in advance, however several (67 %) did occasionally. A majority of both surgeons and the rest of the staff found the surgeons to be refreshed after a pause (Fig. 3).

**Fig. 4:**
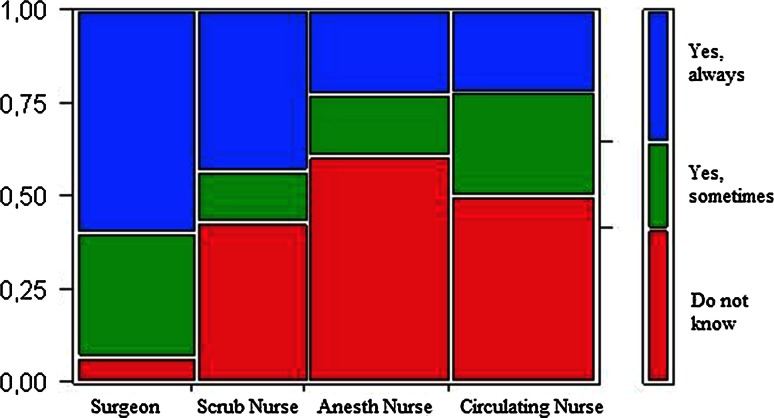
The surgical strategy. Answers in percentages by professional category to the question to surgeons “Has a pause made you change surgical strategy” and to staff “Do you perceive that taking of a pause has made surgeons change surgical strategy?”

Seven (47 %) surgeons said that a pause had made them change their view of the surgical anatomy, which was confirmed by 36 % of the scrub nurses. 60 % of the surgeons correspondingly indicated that pauses had made them change their surgical strategy. Most nurses did not know whether the surgical strategy was changed or not (Fig.  4). Three fourths of the surgeons and scrub nurses assessed that pauses had made the surgeons handle problems in a better way.

A minority (11 %) believed that pauses increased the time in the operating theatre, but a majority of the nurses marked the alternatives of “do not know” or “no change”. This differed somewhat from the surgeons, where several surgeons (40 % each) marked “no change” or decreased time.

A majority (82 %) of the staff suggested that the surgeons should take pauses more often. Surgeons (93 %) and scrub nurses (79 %) experienced that the pause increased patient safety. This was less true for the anaesthetist nurses (67 %) and circulating nurses (39 %). No one answered that they believed that the patient safety decreased. Most nurses believed that the safety issue was ascertained during pauses, even when the surgeons temporarily left the operating theatre.

Notable among the written comments were remarks like “you clearly see the increasing fatigue over time with poor concentration and high irritation”, “you see that the surgeons get new energy, which increase patient safety” and “I would prefer extra door openings to have alert surgeons if I was the patient”. There was no significant change in operative times from before (*m* = 310, SD 98.67, CI −47.8; 11.7) to after (*m* *=* 328, SD 94.38, CI −47.7; 11.7, *p* = 0.233) introduction of the pause routine.

## Discussion

The findings in this study indicate that surgeons appreciate scheduled pauses and that it affects their performance. Engelmann et al. have previously shown interesting results in a randomized study on laparoscopic surgery with clear advantages in taking pauses [22]. The described pause schedule was every half hour, while another study on paediatric surgery by the same group had 25-min intervals [23]. They also indicated that acceptance of the pause was of importance for its effect. During the implementation of our routine, we made an effort to include the team and gain acceptance. The choice of a 2 h limit was an arbitrary balance between the mentioned studies above and the surgical assessment that operations lasting less than 2 h could be safely performed without a pause.

The findings support that the surgeons felt refreshed even after a shorter pause. This was also confirmed by the assessment of the scrub nurses. However, it is interesting that there is a difference in the perception of effect on the surgeon between the scrub nurses and the anaesthetist nurses, and the circulating nurses. A similar pattern was also seen in questions regarding team collaboration. A plausible hypothesis could be the difference in communication between different sub-groups in the team [3]. Dividing the team into two groups, those who scrubbed in work more closely together and the anaesthetist and circulating nurses who are physically a little more distant. This is however worrying as the work in the operating theatre is dependent on a team effort where all members need to be involved closely. It is possible that the surgeons as leaders in the operating theatre need to acknowledge the active participation of all team members. Reduction of practical obstacles in the operating theatre environment such as noise and background sounds could also facilitate the communication and the sense of a team effort. [13, 18].

The finding that surgeons need reminders also concurs with studies of how surgeons perceive their own skills and performance [23]. Surgeons often decline being affected by stress and fatigue during surgery [13, 24]. We believe that the surgeons are a part of the team and need the support of the other members to keep track of time in relation to surgical progression and awareness of increased fatigue. Also, the surgeon should appreciate the team’s feedback on those issues as well as of a possible notification of intraoperative fatigue. Moreover, we suggest the importance of setting good examples for younger surgeons regarding non-technical skills including communication. The thought and environment for setting up the pause might be of more value for fatigue awareness and safety thinking, including openly asking for assistance, than just the physical side.

The different experiences described by surgeons and nurses regarding the assessment of the anatomy or changes in surgical strategy could be explained by lack of communication. This suggests that the surgeons should communicate better with all team members by continuous short updates and teaching to improve procedural understanding and keeping the entire team updated on the progress and difficulties. The results indirectly suggest that surgeons could improve non-technical skills including team leadership through better communication and plausibly also through strategically awareness shown by time and pause planning. Also, as the results indicate that changes in perception and strategy do occur, it might support a conclusion that pauses can promote patient safety.

This study has some limitations. One is the different answer frequency by the professions in the team. Anaesthesia and circulating nurses answered to a lesser degree perhaps due to the organisation in the operating theatre. They are involved to a larger extent than surgeons and scrub nurses in many other procedures without pauses and thus they may not feel as involved. Other limitations are the risk of answer skewing bias related to ones opinion of the routine and the lack of objective data on patient safety. The latter would require another type of study with a large patient material over a long period of time. Further studies could also include semi-objective parameters such as team performance through observation by using the “Observational Teamwork Assessment for Surgery—OTAS” or “The Oxford Non-Technical Skills scale—NOTECHS [25, 26]. One possible hard data could have been operation times. Although there was no significant change, we acknowledge that there are many confounders for time aspects. However, even with long pauses, they constitute <10 % of total times for long procedures where every misstep is an issue of both additional time to resolve and a risk for the patient.

A strength of this study was that the staff answered the questionnaire anonymously to reduce the risk of bias. Another strength was that implementation was well accepted by all surgeons; thus, there was a uniform adherence to the routine.

## Conclusion

The pause routine was well perceived by the surgical team. A majority believed that scheduled and regular pauses contributed to improved patient safety and better team communication. There were also findings of differences in communication and experience of team coherence between personnel categories that could benefit from further acknowledgement and exploration.
